# Pulmonary valve neo-reconstruction using the Ozaki technique in an adapted Ross procedure: case report

**DOI:** 10.1093/ehjcr/ytaf291

**Published:** 2025-06-18

**Authors:** Adrián Kolesár, Štefan Lukačín, Vilém Rohn, Jaroslav Benedík, Tomáš Toporcer

**Affiliations:** Department of Heart Surgery, East Slovak Institute of Cardiovascular Diseases and Medical Faculty, University of Pavol Jozef Šafárik, 04011 Košice, Slovakia; Department of Heart Surgery, East Slovak Institute of Cardiovascular Diseases and Medical Faculty, University of Pavol Jozef Šafárik, 04011 Košice, Slovakia; I. Surgery Department—Cardiovascular Surgery, General University Hospital and Second Faculty of Medicine, Charles University, 12808 Prague, Czech Republic; Department of Cardiac Surgery, Na Homolce Hospital, 15030 Prague, Czech Republic; Department of Heart Surgery, East Slovak Institute of Cardiovascular Diseases and Medical Faculty, University of Pavol Jozef Šafárik, 04011 Košice, Slovakia

**Keywords:** Aortic valve stenosis and regurgitation, Ross procedure, Ozaki procedure, Case report

## Abstract

**Background:**

Aortic valve damage is the most common valvular heart disease in developed countries. The Ross procedure is an alternative to the aortic valve replacement with a prosthesis, providing a longer survival without reoperation and without the need for anticoagulation therapy. The unavailability of homografts for the pulmonary valve replacement is one of the limiting factors for a more common utilization of this therapeutic method.

**Case summary:**

This case report presents a 35-year-old Caucasian male with a bicuspid aortic valve and severe aortic regurgitation. The patient underwent the Ross procedure with the stabilization of the aortic ring and sinotubular junction. A tubular prosthesis was created from the bovine pericardium, into which three neo-cusp valves were fashioned from autologous pericardium. The new pulmonary conduit was sutured distally to the distal pulmonary trunk and proximally to the right ventricular outflow tract. Four months post-operatively, the follow-up echocardiographic examination documented that the valve in the aortic position had no regurgitation, a maximum velocity of 1.25 m/s, and a peak gradient of 6.25 mmHg. The valve in the pulmonary position showed a trace regurgitation and mean pressure gradient of 13 mmHg.

**Discussion:**

The combination of the modified Ross procedure with neo-cuspidalization according to Ozaki for a new pulmonary valve thus offers hope for an extended survival without reoperation in paediatric and non-elderly adult patients with an aortic valve dysfunction. This technique is additionally applicable by cardiac surgery centres without access to pulmonary homografts.

Learning points

- The Ross procedure for treating aortic stenosis or regurgitation in non-elderly patients offers benefits over both mechanical and bioprosthesis.
- The modification of the Ross procedure involving neo-cuspidalization of the pulmonary valve presents a new strategy for centres with limited access to homografts.

## Introduction

Aortic valve damage is the most common valvular heart disease in developed countries. The incidence of aortic valve stenosis in western developed countries is 52.5 newly diagnosed cases per 100 000 adult inhabitants per year.^1^ The gold standard of therapy for both stenotic and insufficient valves, when repair is not possible, is the replacement of the valve with a mechanical or biological valve prosthesis. Mechanical valve prostheses offer a long-term stability, but require lifelong anticoagulation with warfarin. The implantation of a bioprosthesis does not require the use of anticoagulants, but their durability is time-limited. The European Association for Cardio-Thoracic Surgery guidelines recommend a bioprosthesis implantation in patients over 65 years of age and the use of a mechanical prosthesis in patients under 60 years of age.^2^ Neither of these prostheses offers an optimal therapy for non-elderly patients.

The search for an ideal substitute for the aortic valve led Donald Ross to develop the pulmonary autograft concept in 1967.^3^ The Ross procedure involves replacing the aortic valve with a pulmonary autograft.^4^ The gold standard is the replacement of the pulmonary valve with a homograft from a deceased donor. As an alternative replacement, bovine jugular veins or xenografts are mentioned in the literature.^4,5^ The Ross procedure is associated with several advantages in non-elderly adults in terms of haemodynamics, valve-related complications, and survival. However, the homograft and its durability represent important parameters affecting survival without reoperation.^6^ Moreover, the availability of homografts depends on the number of suitable donors, and it is associated with high storage costs.

The first attempts to replace the leaflets of the aortic valve with *fascia lata* or pericardium date back to the 1960s.^7^ The first larger group of patients (*n* = 404) was published by Ozaki in 2011.^8^ The Ozaki’s technique involves the replacement of degenerated aortic valve leaflets with pericardial tissue treated with glutaraldehyde (GA). In the middle of the post-operative period, the technique demonstrates very promising haemodynamic results.

Recent studies present the use of neo-cuspidalization with the Ozaki technique as a possible alternative for the pulmonary valve reconstruction due to endocarditis.^9^ The neo-cuspidalization of the pulmonary valve using autologous pericardium in the modified Ross procedure has been chosen as a new therapeutic strategy for aortic stenosis and regurgitation.

## Summary figure

**Figure:**
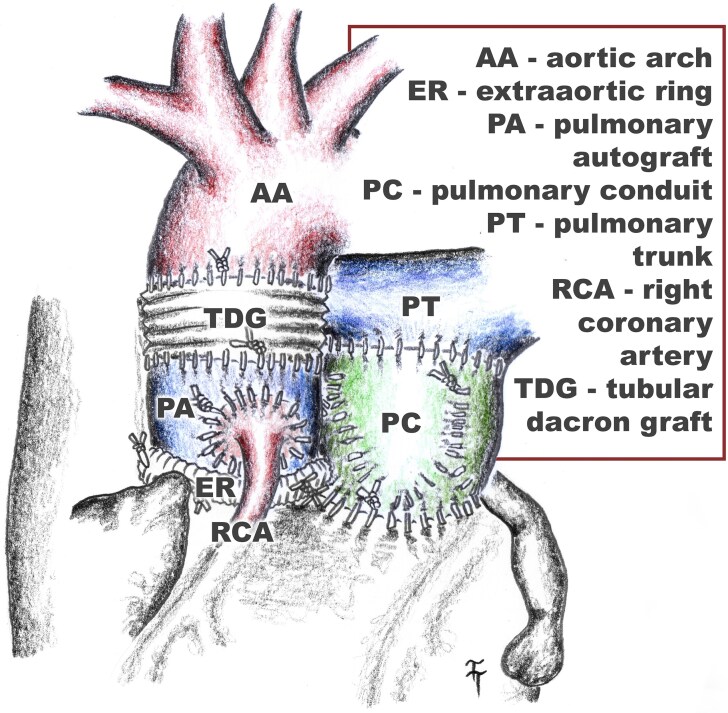


## Case presentation

**Figure 1:**
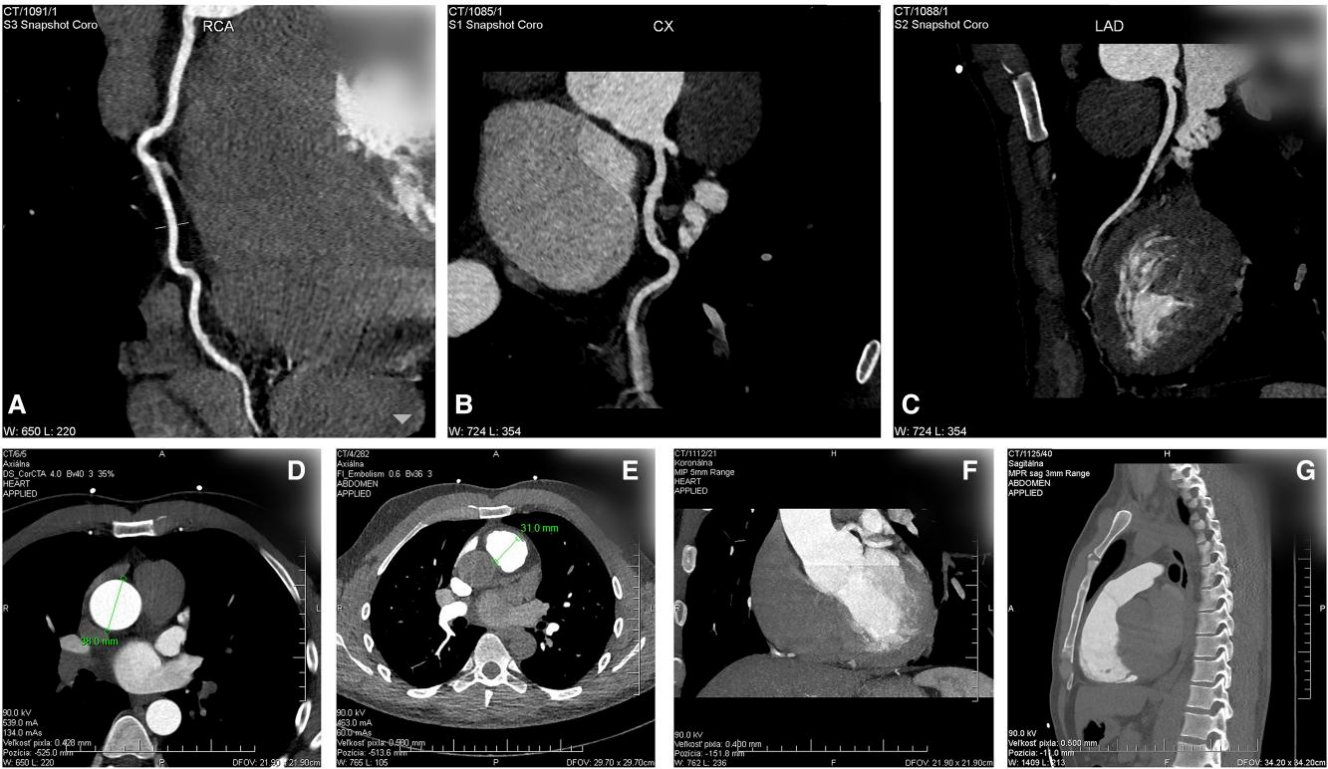
Computed tomography images focused on the anatomy of the coronary tree and the diameters of the aorta and pulmonary artery: (*A*) right coronary artery; (*B*) circumflex artery; (*C*) left anterior descending artery; (*D*) aorta diameter; (*E*) pulmonary artery diameter; (*F*) anatomy of the aorta; (*G*) anatomy of the pulmonary artery.

**Figure 2:**
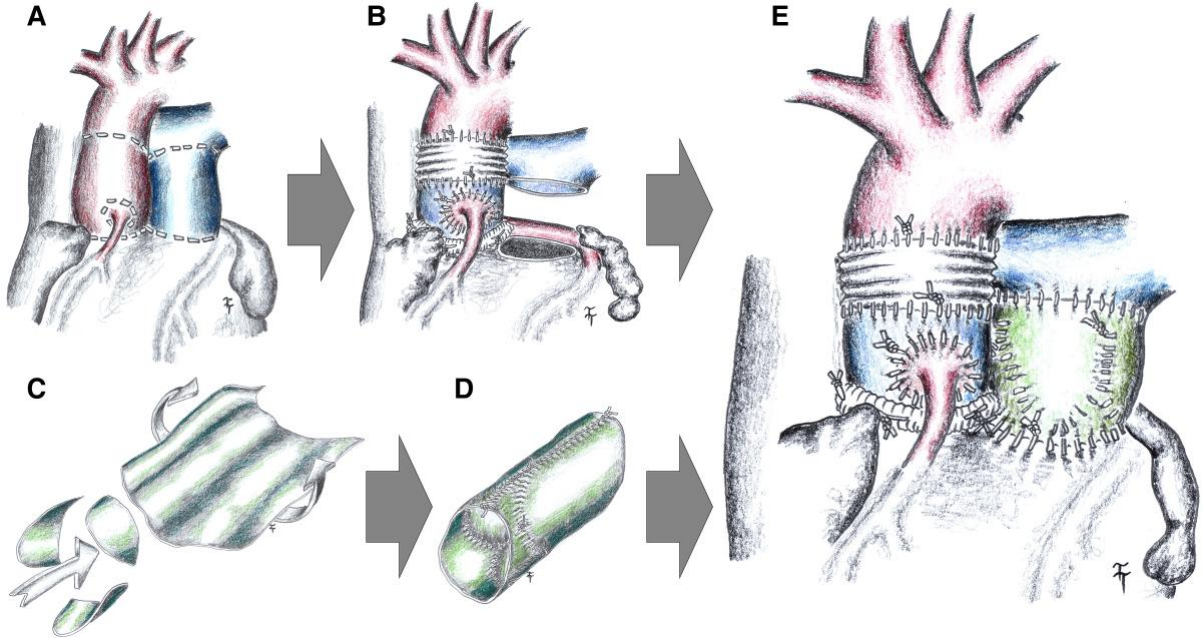
Sketch of the surgery: (*A*) resection lines on the aorta and pulmonary artery; (*B*) pulmonary autograft implanted in the aortic position; (*C* and *D*) creation of a pulmonary artery from the bovine pericardium and a pulmonary valve using the Ozaki method from autologous pericardium; (*E*) status post-surgery; red—tissues from the systemic circulation; blue—tissues from the pulmonary circulation; green—tissues from autologous and xenologous pericardium.

This case report was conducted in accordance with the Declaration of Helsinki and was approved by the Ethics Committee of the corresponding author’s institution (protocol code A1102024; date of approval: 15 October 2024). The informed consent with hospitalization and surgery was obtained from the patient and is available for the editor. A 35-year-old Caucasian male with bicuspid aortic valve (Type 1/LR) and severe aortic regurgitation (effective regurgitant orifice area: 0.38 cm²) was admitted for surgery. The patient exhibited a diastolic murmur, and all screening laboratory tests were within normal limits. The patient showed no signs of acute heart failure and, due to the valvular defect, has been managed with long-term therapy. The patient had no other valvular defects, and the left ventricular function was normal [left ventricular ejection fraction (LVEF): 59%, left ventricular end-systolic diameter (LVESD): 41 mm, left ventricular end-diastolic diameter (LVEDD): 60 mm]. Computed tomography did not demonstrate any coronary artery stenosis. The pulmonary trunk had dimensions of 26 × 39 mm at the height of the valve and 25 × 36 mm above the valve. The aorta measured 27 × 35 mm at the height of the valve annulus and 40 × 43 mm at the level of the aortic root (*Figure 1*). The patient declined a mechanical aortic valve prosthesis, and pulmonary homografts were unavailable at our centre at the time of patient management. Therefore, the patient was indicated for modified Ross surgery, while the neo-valve creation method according to Ozaki was chosen as the replacement of the pulmonary valve. Under general anaesthesia after medial sternotomy, a sufficiently large pericardium was first removed and treated with GA (2.5% GA solution for 10 min, followed by three washes in saline, for 2 min each). The patient was connected to cardiopulmonary bypass (CPB) in a standard way using the bi-stage venous cannula. After administering the cardioplegic solution, the aortic valve and aortic root were explanted. In order to stabilize the aortic annulus, a 34 mm extra-aortic ring was implanted. After the preparation of the pulmonary autograft, the graft was implanted in the aortic position using individual sub-annular sutures. The coronary arteries were implanted into the autograft. The stabilization of ST junction was performed by interposition of tubular Dacron graft with the size of 28 mm. Finally, the distal anastomosis between the Dacron graft and distal aorta has been done. A tubular prosthesis was created from the bovine pericardium (size of 9 × 14 cm), into which three neo-cusp valves were created from the autologous pericardium (see Supplementary material online, *Video S1*). The new pulmonary conduit was sutured distally to the distal pulmonary trunk and proximally to the right ventricular outflow tract (RVOT) (*Figure 2*) (see Supplementary material online, *Video S2*). The length of CPB was 248 min, and the length of the aortic clamp was 209 min. Post-operatively, no significant complications were noted. Post-operative echocardiography documented the perfect function of the autograft (Vmax: 1.03 m/s; PG max 6 mm of Mercury column) and the neo-valve in the position of the pulmonary valve (Vmax: 1.32 m/s). A trace regurgitation of constructed pulmonary valve and no regurgitation of the valve in aortic position were documented before discharge. The patient was discharged on the 14th post-operative day.

## Discussion

The Ross procedure represents a significant benefit for the patient in terms of durability and resistance to structural deterioration compared to a biological valve.^10^ In the short-term follow-up, the benefits also surpass those of mechanical valve prostheses in terms of survival.^11^ In the long term, the Ross procedure is associated with a higher risk of reoperations compared to mechanical prostheses due to a structural degeneration of both the autograft and the homograft.^10^ Twenty years after the classical Ross procedure, the probability of pulmonary homograft dysfunction is 19.7%. This is a 1.5 times higher incidence compared to the risk of autograft failure.^12^ Several modifications of the homograft, such as decellularization, have been tried to extend its durability.^6^ The use of a bovine jugular vein or xenograft shows a similar risk of reintervention because of graft failure over a 15-year follow-up period.^5^ However, the best way to avoid an immune response to the homograft is to use an autologous tissue.

Neo-cuspidalization according to Ozaki in the aortic position, compared to the bovine stented aortic valve replacement, presents the same reoperation risk over a 6-year follow-up period, but shows statistically lower peak gradients. By using the Ozaki technique, we can avoid an early prosthetic failure observed in certain models of biological valve replacements.^13^ Recently, several modifications of the Ozaki technique used in the aortic position have been published. These techniques, in addition to replacing the aortic valve leaflets with autologous or xenologous pericardium, also replace the aortic root, usually with a vascular prosthesis. This includes the presentation of the Bentall procedure with the implantation of a new aortic valve constructed using the Ozaki technique.^14^ The use of the Ozaki technique for pulmonary valve reconstruction in cases of stenosis has also been described.^15^ All these modifications show excellent early post-operative results. However, long-term follow-ups have not been published.

The combination of the modified Ross procedure with neo-cuspidalization according to Ozaki for a new pulmonary valve thus offers hope for an extended survival without reoperation in paediatric and non-elderly adult patients with an aortic valve dysfunction. This technique is additionally applicable in/by cardiac surgery centres without access to pulmonary homografts.

## Patient perspective

Four months after the operation, the patient is well. Echocardiographic examination documents a good left ventricular function with an LVEF of 60%, LVESD of 40 mm, and LVEDD of 52 mm and a good right ventricular function as well. The valve in the aortic position has no regurgitation, a maximum velocity of 1.25 m/s, and a peak gradient of 6.25 mmHg. The valve in the pulmonary position shows a trace regurgitation and a mean pressure gradient of 13 mmHg.

At the time of publication, a total of four patients had been operated on using the aforementioned technique at the corresponding authors’ institution.

## Supplementary Material

ytaf291_Supplementary_Data

## Data Availability

The data underlying this article are available in the article and in its online Supplementary material.

## Lead author biography

Assoc. Prof. MD Adrián Kolesár, PhD, MPH, is the head of the Department of Heart Surgery at the Medical Faculty of Pavol Jozef Šafárik University. His surgical practice focuses on aortic valve-sparing procedures. He is a member of the European Association for Cardio-Thoracic Surgery (EACTS) and national professional organizations.
